# OzEAN Study to Collect Real-World Evidence of Persistent Use, Effectiveness, and Safety of Ozanimod Over 5 Years in Patients With Relapsing-Remitting Multiple Sclerosis in Germany

**DOI:** 10.3389/fneur.2022.913616

**Published:** 2022-06-27

**Authors:** Tjalf Ziemssen, Stephan Richter, Mathias Mäurer, Mathias Buttmann, Boris Kreusel, Anne-Maria Poehler, Maren Lampl, Ralf A. Linker

**Affiliations:** ^1^Department of Neurology, Center of Clinical Neuroscience, Carl Gustav Carus University Clinic, University Hospital of Dresden, Dresden, Germany; ^2^Mind MVZ, Center of Neurology, Stuttgart, Germany; ^3^Department of Neurology, Klinikum Würzburg Mitte, Würzburg, Germany; ^4^Department of Neurology, Caritas Hospital Bad Mergentheim, Bad Mergentheim, Germany; ^5^Bristol Myers Squibb GmbH & Co. KGaA, Munich, Germany; ^6^Department of Neurology, University Hospital of Regensburg, Regensburg, Germany

**Keywords:** relapsing-remitting multiple sclerosis, observational study, real-world evidence, patient-reported outcomes, protocol, trial-in-progress, medication adherence, medication persistence

## Abstract

**Background:**

Ozanimod, a sphingosine 1-phosphate receptor 1 and 5 modulator, was approved as a disease-modifying therapy for active relapsing-remitting multiple sclerosis (RRMS) in 2020 and for active ulcerative colitis in 2021. Long-term, real-world studies in a nonselective population are needed. OzEAN is an ongoing study to assess the real-world persistent use, effectiveness, and safety of ozanimod and its impact on quality of life (QoL) in patients with RRMS over a 5-year period.

**Methods:**

This prospective, noninterventional, postmarketing authorization study will enroll ~1,300 patients (≥18 years of age) with active RRMS. The decision to initiate ozanimod must have been made before and independent from study participation. Enrollment began in March 2021. Recruitment is ongoing and will last for 36 months across 140 sites in Germany. Treatment-naive patients or those having prior experience with a disease-modifying therapy receive oral ozanimod 0.92 mg/day after an initial dose escalation, per the summary of product characteristics recommendations, for up to 60 months. Persistence with ozanimod treatment (primary endpoint) is assessed at month 60. Secondary endpoints include additional physician-reported outcomes [persistence at earlier time points, annualized relapse rate, Expanded Disability Status Scale score, cognition (Symbol Digit Modalities Test), and incidence of adverse events], and patient-reported outcomes assessing patient satisfaction, adherence, and treatment modalities (Treatment Satisfaction Questionnaire for Medication, v1.4), disability (United Kingdom Neurological Disability Rating Scale), QoL (MSQOL-54 questionnaire), fatigue (Fatigue Scale for Motor and Cognitive Functions), and health economics [Work Productivity and Activity Impairment Questionnaire for Multiple Sclerosis (German v2.1); Multiple Sclerosis Health Resource Survey, v3.0]. A Multiple Sclerosis Documentation System with an internet-based e-health portal allows patients to view files and complete questionnaires. A safety follow-up will occur 3–8 months after the last ozanimod dose for patients who discontinue treatment early. Long-term results are anticipated after study completion in 2029. Yearly interim analyses are planned after enrollment has reached 25%.

**Conclusion:**

This is the first long-term, real-world study of ozanimod in patients with RRMS and, to our knowledge, the first noninterventional study utilizing a patient portal. These data will add to the safety/efficacy profile of ozanimod demonstrated in phase 3 trials.

**Clinical Trial Registration:**

Clinicaltrials.gov, identifier: NCT05335031.

## Introduction

Ozanimod is a sphingosine 1-phosphate receptor 1 and 5 modulator that blocks lymphocyte egress from lymphoid tissue, reducing the number of circulating lymphocytes in peripheral blood (1). Ozanimod was first approved in the United States in 2020 for the treatment of adults with relapsing forms of multiple sclerosis (RMS) and subsequently approved in multiple countries for adults with active relapsing-remitting multiple sclerosis (RRMS) defined by clinical or imaging results; in 2021, it was approved for the treatment of moderately to severely active ulcerative colitis in the United States and European Union (2, 3).

**Figure 1:**
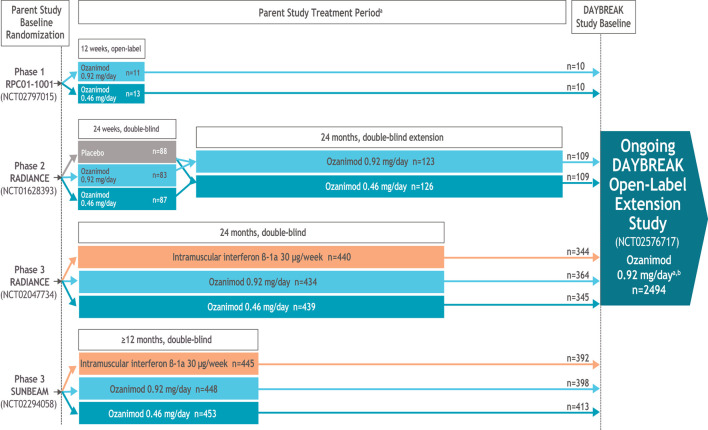
Ozanimod clinical development in RMS program. ^a^In all trials, upon initiation of ozanimod, patients received 0.23 mg on days 1–4, 0.46 mg on days 5–7, and then their assigned dose of 0.46 or 0.92 mg on day 8 and thereafter. All patients entering the phase 2 dose-blinded extension period underwent dose escalation, even if treated with ozanimod in the parent trial, to maintain the blind. ^b^In DAYBREAK, dose escalation was performed for all patients entering from one of the active-controlled phase 3 trials, irrespective of prior treatment assignment (to maintain the blinding in the parent trials); dose escalation was not performed for those entering from the phase 1 or 2 trials, unless the last dose of ozanimod was >14 days before entering DAYBREAK.

The ozanimod clinical development program in RMS (Figure 1) included a phase 1 pharmacokinetic/ pharmacodynamic trial (12 weeks), a phase 2 placebo-controlled trial (24 weeks) with a dose-blinded extension (24 months) (4, 5), and two phase 3 active-controlled trials, RADIANCE (24 months) (6) and SUNBEAM (minimum 12 months) (7). In both phase 3 trials, ozanimod 0.92 mg/day was associated with lower adjusted annualized relapse rate (ARR), fewer gadolinium-enhancing lesions and new or enlarging T2 lesions on brain magnetic resonance imaging (MRI), and reduced brain volume loss compared with intramuscular interferon β-1a 30 μg weekly (6, 7). The most frequent adverse events (AEs) associated with ozanimod treatment were upper respiratory infection, hepatic transaminase elevation, orthostatic hypotension, urinary tract infection, back pain, and hypertension. A pooled analysis of safety results from all trials, including an ongoing open-label extension study (DAYBREAK), were consistent with those of the phase 3 trials and demonstrated no new safety concerns (8). Patients in the parent trials were adults (18–55 years of age) with multiple sclerosis (MS) [diagnosed per 2010 McDonald criteria (9)] with a relapsing clinical course, brain MRI lesions consistent with MS, an Expanded Disability Status Scale (EDSS) (10) score of 0–6.0 (phase 1) or 0–5.0 (phase 2 and 3), and a history of relapses within the past 1–2 years (phase 2 and 3) (4, 6, 7).

Given the recent approvals of ozanimod, long-term studies in a non-selective, real-world population are not yet available, but are needed to evaluate ozanimod in a broader population of patients. Such studies could also provide information on persistence and adherence with ozanimod treatment, disease characteristics and treatment history of patients who are prescribed ozanimod, and patient-reported outcomes and pharmacoeconomic data, outcomes that were not evaluated in the clinical development program.

OzEAN is an ongoing prospective, non-interventional, postmarketing authorization observational cohort study to assess real-world persistent use, effectiveness, and safety of ozanimod, and the impact of treatment on quality of life (QoL), in patients with RRMS in Germany over a 5-year period. These real-world data, including patient-reported QoL outcomes, are of interest to German authorities and support their Health Technology Assessment (11).

## Methods and Analysis

### Study Setting and Treatment

**Figure 2:**
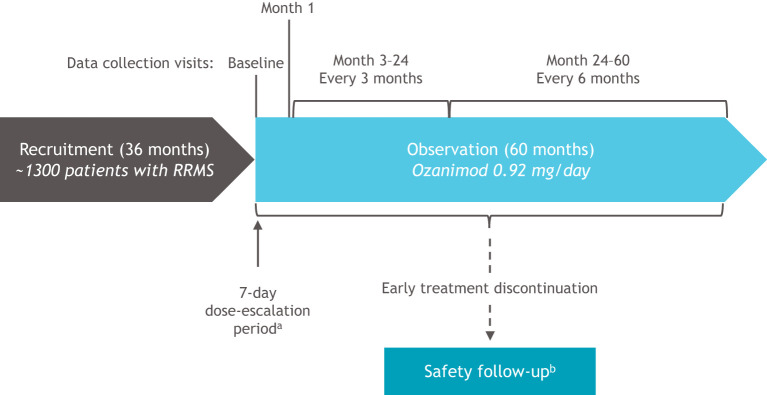
OzEAN study design. MS, multiple sclerosis; RRMS, relapsing-remitting multiple sclerosis. ^a^Days 1–4: ozanimod 0.23 mg; days 5–7: ozanimod 0.46 mg; days 8 and thereafter: ozanimod 0.92 mg. ^b^Patients who discontinue ozanimod prior to end of study (month 60) will be withdrawn from the OzEAN study and a safety follow-up will be performed 3–8 months after the last dose of ozanimod when a new therapy for MS is started or after 8 months (at the latest) if no subsequent MS therapy is initiated.

Eligible patients will be enrolled in study sites across Germany. The decision to initiate ozanimod treatment must have been made by the physician before and independent from enrollment into the study. Patients receive oral ozanimod 0.92 mg/day for up to 60 months following a 7-day dose escalation regimen according to the summary of product characteristics (SmPC) (3). During this observation period, patients are evaluated at 16 data collection visits: baseline (visit 1), month 1 (visit 2), quarterly from month 3 to month 24 (visits 3–10), and at 6-month intervals from month 24 to month 60 (visits 11–16; Figure 2). Patients who permanently discontinue ozanimod treatment before month 60 are withdrawn from the observational study. A safety follow-up is performed when a new MS therapy is initiated (3–8 months after receiving the final dose of ozanimod) or 8 months (at the latest) after receiving the final dose of ozanimod if no new MS therapy is initiated.

The individual study duration (observation period) per patient includes up to 60 months for the noninterventional treatment documentation period, and approximately 3–8 months for the safety follow-up period in case of premature discontinuation of ozanimod. The overall study duration is 104 months, including a 36-month recruitment period, 60-month observational period, and up to 8-month safety follow-up period. The entire study period is planned to last from the enrollment of the first patient (March 2021) until March 2029.

### Eligibility Criteria

Eligible patients are adults (≥18 years of age) diagnosed with RRMS who are either treatment-naive or have prior experience with a disease-modifying therapy and elected to switch to ozanimod. Patients with contraindications specified in the current version of the SmPC (3), or with known hypersensitivity to ozanimod or any of its excipients, are excluded. Patients may not be participating in any other clinical studies.

Inclusion and exclusion criteria are shown in Table 1.

**Table 1:** Eligibility criteria.

**Inclusion Criteria**	**Exclusion Criteria**
Males or females aged ≥ 18 years who provided written informed consent	Contraindications specified in the current version of the SmPC
Confirmed diagnosis of RRMS according to ICD-10 and eligible for treatment with ozanimod according to physician's judgement and based on the recommendation of the current SmPC	Known hypersensitivity to the active substance(s) or to any of the excipients of ozanimod as specified in the SmPC
The decision to initiate treatment with ozanimod must have been made by the treating physician before enrollment and independently of this study; retrospective documentation of ozanimod therapy and enrollment of patients that are already on ozanimod therapy is not allowed	Participation in any other clinical studies

*ICD-10, International Statistical Classification of Diseases and Related Health Problems, 10^th^ version; RRMS, relapsing-remitting multiple sclerosis; SmPC, Summary of Product Characteristics*.

### Endpoints and Assessments

At baseline, the following data are collected: patient demography, vital parameters, physical status, prior and concomitant diseases and medications, MS diagnosis, MS history (including prior relapses), prior MS treatment, reason for initiating/switching to ozanimod, and treatment modalities with ozanimod. In addition, physician-reported outcomes [EDSS (10) and Symbol Digit Modalities Test (SDMT) (12, 13)] and patient-reported outcomes (PROs; Treatment Satisfaction Questionnaire for Medication, version 1.4 (TSQM v1.4) (14, 15), United Kingdom Neurological Disability Rating Scale (UKNDS) (16, 17), Multiple Sclerosis Quality of Life Instrument-54 items (MSQOL-54) (18), Fatigue Scale for Motor and Cognitive Functions (FSMC) (19), Work Productivity and Activity Impairment Questionnaire for Multiple Sclerosis, version 2.1 (WPAI-MS v2.1) (20), and Multiple Sclerosis Health Resource Utilization Survey, version 3.0 (MS-HRS v3.0) (21) are completed. Following the baseline visit, these physician-reported outcomes and PROs, together with physician-reported clinical relapses (the occurrence of new symptoms or the worsening of old symptoms) are completed at 3-, 6-, or 12-month intervals during the 60-month observation period (Table 2). Alternate forms of the SDMT will be used to avoid practice effects. The following characteristics of ozanimod treatment are documented continuously: initial dose escalation; maintenance dose; temporary interruptions of treatment, including date and reason for interruption, and re-initiation of therapy following treatment interruption; and all medication taken concomitantly with ozanimod and all changes in concomitant medication during the study, including reason for administration. Primary and secondary endpoints are summarized in Table 3.

**Table 2:** Schedule of enrollment, interventions, and assessments.

**Assessment/Data Collected**	**Observation**				**End of observation^a^**	**Safety follow-up^b^**
	**Enrollment**	**Month 1**	**Month 3–24**	**Month 24–60**		
						**Last treatment +**
	**Baseline**		**Every 3 months**	**Every 6 months**	**End of study**	**3–8 months**
**Baseline**						
Informed consent	X					
Inclusion/exclusion criteria	X					
Demography	X					
General medical history	X					
MS history and pretreatment^c^	X					
Concomitant diseases	X					
All previous malignant diseases	X					
All other previous diseases within 5 years	X					
prior to study enrollment
Physical status^d^	X					
**Treatment**						
Treatment modalities^e^	X	X	X	X		X
Concomitant medication	X	X	X	X		X
Subsequent MS treatment					X	X
**Physician-reported assessment**						
Persistence with therapy	Assessed continuously throughout study					
Clinical relapse (ARR)		X	X^f^	X^f^		X
EDSS	X	X	X^f^	X^f^		X
SDMT	X	X^g^	X^g^	X		X
MRI^h^	X^h^	X^h^	X^h^	X^h^		X^h^
**Patient-reported assessment**						
Adherence to therapy^i^	X^i^	X^i^	X^i^	X^i^	X^i^	X^i^
TSQM v1.4	X	X	X^j^	X		X
UKNDS	X	X	X^g^	X		X
MSQOL-54	X	X	X^g^	X		X
FSMC	X	X	X^g^	X		X
WPAI-MS v2.1	X	X	X^g^	X		X
MS-HRS v3.0	X	X	X^g^	X		X
**Safety assessment**						
AEs/SAEs	Assessed continuously throughout study					
Laboratory panel^h^	X^h^	X^h^	X^h^	X^h^		X^h^

*AE, adverse event; ARR, annualized relapse rate; EDSS, Expanded Disability Status Scale; FSMC, Fatigue Scale for Motor and Cognitive Functions; MRI, magnetic resonance imaging; MS, multiple sclerosis; MSFC, Multiple Sclerosis Functional Composite; MS-HRS v3.0, Multiple Sclerosis Health Resource Survey, version 3.0; MSQOL-54, Multiple Sclerosis Quality of Life-54; SAE, serious adverse event; SDMT, Symbol Digit Modalities Test; TSQM v1.4, Treatment Satisfaction Questionnaire for Medication, version 1.4; UKNDS, United Kingdom Neurological Disability Rating Scale; WPAI-MS v2.1, Work Productivity and Activity Impairment Questionnaire for Multiple Sclerosis, version 2.1*.

a *Documentation performed directly after a patient reached the regular end of study (at month 60) or permanently discontinued ozanimod treatment before month 60; if routine data were collected that do not meet any of the documentation time points offered, these data may be entered with the month 60 documentation*.

b *Follow-up documentation of potential AEs and subsequent therapy, performed approximately 3–8 months after stopping treatment with ozanimod and when a new MS therapy is initiated, or after 8 months (at the latest) if there is no subsequent MS therapy initiated*.

c *Includes MS diagnosis according to International Classification of Diseases, Tenth Revision; first manifestation of MS; course of MS disease and number of relapses within the year before enrollment; and type and duration of prior disease-modifying therapies for MS*.

d *Includes vital parameters, physical status*.

e *Treatment modalities referring to ozanimod, including (planned) date of first administration of ozanimod, reason for switch to ozanimod, interruptions, re-initiation of therapy following treatment interruption, and reason for discontinuation in case of switch to another MS treatment*.

f *At yearly intervals (months 12, 24, 36, 48, and 60) only*.

g *At 6-month intervals (months 6, 12, 18, and 24) only*.

h *Only if available based on the local clinical routine assessments performed at the study center: MRI (number of lesions) and relevant laboratory measurements, especially those for monitoring ozanimod therapy (e.g., liver parameters, such as alanine aminotransferase, aspartate aminotransferase, gamma-glutamyl transferase, and total bilirubin; differential blood count; or lymphocyte count)*.

i *Patient-reported qualitative assessment of how often the patient missed doses and how regularly he or she took the medication, by entering the date of starting with a new ozanimod package and selection of the package size. This information is collected electronically via the patient portal application or, alternatively, using paper-based questionnaires provided at the local study center*.

j *At 3-month intervals (month 3, 6, 9, 12, 15, 18, 21, and 24)*.

**Table 3:** Primary and secondary endpoints in the OzEAN study.

**Domain**	**Reported by**	**Assessment**	**Outcome measure**	**Time point**
**Primary endpoint**				
Treatment satisfaction	Physician	Persistence with therapy	• Proportion of patients who remain on continuous treatment with ozanimod • Evaluated at month 60	Collected continuously
**Secondary endpoints**				
Treatment satisfaction	Physician	Persistence with therapy	• Proportion of patients who remain on continuous treatment with ozanimod • Evaluated at months 12, 24, 36, and 48	Collected continuously
	Patient	Adherence to therapy	• Percentage of dose taken as prescribed • Evaluated at months 12, 24, 36, 48, and 60	Months 3, 6, 9, 12, 15, 18, 21, 24, 30, 36, 42, 48, 54, and 60, and SFU
	Patient	TSQM v1.4	• Changes in TSQM v1.4 domains • Relationship between each TSQM v1.4 domain and clinical outcomes^a^	Baseline and months 3, 6, 9, 12, 15, 18, 21, 24, 30, 36, 42, 48, 54, and 60, and SFU
Effectiveness	Physician	Clinical relapse	• Annualized relapse rate	Months 12, 24, 36, 48, and 60, and SFU
	Physician	EDSS	• Change from baseline in EDSS	Baseline and months 12, 24, 36, 48, and 60, and SFU
	Patient	UKNDS	• Change from baseline in UKNDS sum score • Proportion of patients with a clinically meaningful improvement/worsening of 1 grade in each subscale	Baseline and months 6, 12, 18, 24, 30, 36, 42, 48, 54, and 60, and SFU
Cognitive processing speed	Physician	SDMT	• Change from baseline in SDMT • Proportion of patients with: ° Increase in raw score of ≥ 4 points or 10% from baseline (improved) ° Decline in raw score of ≥ 4 points or 10% from baseline (worsened) ° Raw score change from baseline who do not meet improved or worsened definition (stable)	Baseline and months 6, 12, 18, 24, 30, 36, 42, 48, 54, and 60, and SFU
QoL	Patient	MSQOL-54	• Change from baseline in PCS and MCS • Proportion of patients with: ° Increase of ≥ 5 points in PCS and/or MCS (improved) ° Decline of ≥ 5 points in PCS and/or MCS (worsened)	Baseline and months 6, 12, 18, 24, 30, 36, 42, 48, 54, and 60, and SFU
Fatigue	Patient	FSMC	• Change from baseline in FSMC sum score and physical and cognitive subdomains • Proportion of patients with: ° Decline of ≥ 10 points in sum score (improved) ° Increase of ≥ 10 points in sum score (worsened) ° Decline of ≥ 6 points or ≥ 5 points in cognitive and/or physical domain, respectively (improved) ° Increase of ≥ 6 points or ≥ 5 points in cognitive and/or physical domain, respectively (worsened)	Baseline and months 6, 12, 18, 24, 30, 36, 42, 48, 54, and 60, and SFU
Health economics	Patient	WPAI-MS v2.1	• Change from baseline in WPAI-MS v2.1 domains	Baseline and months 6, 12, 18, 24, 30, 36, 42, 48, 54, and 60, and SFU
	Patient	MS-HRS v3.0	• Resource use/direct and indirect costs	Baseline and months 6, 12, 18, 24, 30, 36, 42, 48, 54, and 60, and SFU
Safety	Physician	Incidence rate for AEs^b^	• Number of new cases per population at risk over the follow-up period (per person-time) • Number of patients with event	Collected continuously

*AE, adverse event; EDSS, Expanded Disability Status Scale; FSMC, Fatigue Scale for Motor and Cognitive Functions; MCS, mental health composite summary; MS-HRS v3.0, Multiple Sclerosis Health Resource Survey, version 3.0; MSQOL-54, Multiple Sclerosis Quality of Life-54; PCS, physical composite summary; SDMT, Symbol Digit Modalities Test; TSQM v1.4, Treatment Satisfaction Questionnaire for Medication, version 1.4; UKNDS, United Kingdom Neurological Disability Rating Scale; QoL, quality of life; SFU, safety follow-up; WPAI-MS v2.1, Work Productivity and Activity Impairment Questionnaire for Multiple Sclerosis, version 2.1*.

a *If effect size Cohen's d (used to indicate the standardized difference between 2 means) > 0.3*.

b *Based on the first occurrence of event during the follow-up period*.

The primary endpoint is persistence with therapy (as reported by physicians), defined as the proportion of patients who remain on continuous treatment with ozanimod (with gaps of ≤ 90 days allowed), from baseline to month 60. Persistence from baseline to months 12, 24, 36, and 48 is a secondary endpoint. Adherence to therapy, defined as the percentage of ozanimod doses taken as prescribed (as reported by patients) (22), at months 3, 6, 9, 12, 15, 18, 21, 24, 30, 36, 42, 48, 54, and 60, and at the safety follow-up, is a secondary endpoint.

Additional secondary endpoints include the following physician-reported outcomes: clinical relapse, expressed as ARR at months 12, 24, 36, 48, and 60, and at the safety follow-up (total number of relapses experienced by all patients in this study divided by the total number of days in the study for the patients, and the ratio multiplied by 365); disability, assessed as change in EDSS score from baseline to months 12, 24, 36, 48, and 60, and at the safety follow-up; and cognitive processing speed, measured using the SDMT and quantified as change from baseline in SDMT score, proportion of patients with increase (improvement) or decrease (worsening) in SDMT raw score of ≥ 4 points or 10% from baseline, and proportion of patients with change in SDMT raw score that does not meet criteria for improvement or worsening (stable) at months 6, 12, 18, 24, 30, 36, 42, 48, 54, and 60, and at the safety follow-up.

Secondary endpoints reported by patients (i.e., PROs) include measures of treatment satisfaction, effectiveness, QoL, fatigue, and health economics. Treatment satisfaction is assessed using the TSQM v1.4; secondary endpoints are changes in TSQM v1.4 domains (effectiveness, side effects, convenience, and global satisfaction) and the relationship between each domain and clinical outcomes at months 3, 6, 9, 12, 15, 18, 21, 24, 30, 36, 42, 48, 54, and 60, and at the safety follow-up. Disability is assessed using the UKNDS; secondary endpoints are the change from baseline in UKNDS sum score and the proportion of patients with clinically meaningful improvement or worsening of at least 1 grade in each UKNDS subscale at months 6, 12, 18, 24, 30, 36, 42, 48, 54, and 60, and at the safety follow-up. QoL is assessed using the MSQOL-54; secondary endpoints are change from baseline in the physical composite summary (PCS) and mental health composite summary (MCS) scores, and the proportion of patients with a clinically meaningful change [increase (improvement) or decrease (worsening) of ≥5 points] (23) in PCS or MCS score, at months 6, 12, 18, 24, 30, 36, 42, 48, 54, and 60, and at the safety follow-up. Fatigue is assessed using the FSMC; secondary endpoints include change from baseline in FSMC sum score, physical subscale, and cognitive subscale; proportion of patients with decrease (improvement) or increase (worsening) of ≥10 points in the sum score; proportion of patients with decrease (improvement) or increase (worsening) of ≥6 points in the cognitive domain; and proportion of patients with decrease (improvement) or increase (worsening) of ≥5 points in the physical domain, at months 6, 12, 18, 24, 30, 36, 42, 48, 54, and 60, and at the safety follow-up. Measures of health economics include the WPAI-MS v2.1 and the MS-HRS v3.0; secondary endpoints are the change from baseline in WPAI-MS v2.1 domains at months 6, 12, 18, 24, 30, 36, 42, 48, 54, and 60, and at the safety follow-up, and resource use and direct and indirect costs assessed using the MS-HRS v3.0 at baseline and months 6, 12, 18, 24, 30, 36, 42, 48, 54, and 60, and at the safety follow-up.

Incidence of AEs [number of new cases per population at risk over the follow-up period (per person-time) and number of patients with event] is also a secondary endpoint. AEs are defined as any untoward medical occurrence, which does not necessarily have a causal relationship with treatment.

### Sample Size and Recruitment

Planned recruitment is approximately 1,300 patients. Recruitment is ongoing and will occur over 36 months. Enrollment began in March 2021.

To increase representativeness of selected sites, a large number of participating sites (up to 140) of different types (office- and hospital-based sites specialized in neurology), which are located geographically across Germany, are planned. To discourage physicians from selecting specific patients for inclusion in the study, they are instructed and trained to ask all eligible patients consecutively for their participation.

It is assumed that the persistence at year 5 will be 55% overall, including 50% of patients who switched to ozanimod treatment from another MS treatment and 55%−60% of treatment naive patients. A sample size of 1,331 patients is required to cover the estimated persistence rate of 0.55 with a 2-sided 95% confidence interval (CI) of 0.03 in each direction using the large sample normal approximation and after considering a dropout rate of 15%. This interval width is considered appropriate for correct description of the actual persistence rate on a descriptive level.

### Data Collection, Management, and Analysis

#### Data Collection Methods

**Figure 3:**
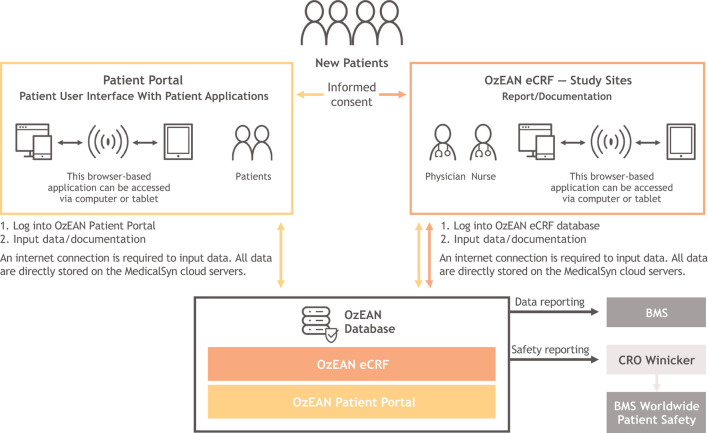
Architecture and data flow within OzEAN eCRF and OzEAN patient portal. Physician-reported assessments are performed at the study site, and data are entered by physicians into the OzEAN eCRF. Patient-reported assessments are completed by patients using the OzEAN patient portal, and data are fed into the OzEAN eCRF. The patient portal interface additionally provides a calendar, medication plan, documents module, and access to surveys (PROs). Data from the OzEAN eCRF are transmitted to Winicker Norimed GmbH (the contract research organization) for storage on secure servers. Data on adverse events are additionally transmitted to the sponsor or its designee as part of standard safety reporting. CRO, contract research organization; eCRF, electronic case report form; PRO, patient-reported outcome.

As part of routine care, the study physician or qualified study staff members at the study site enter data on treatment, MS relapses, EDSS, SDMT, and AEs since the patient's last visit, according to the timeline outlined in Table 2, using an electronic case report form (eCRF; Figure 3). Laboratory panels and MRIs are performed only as available based on routine clinical assessments at each study center.

**Figure 4:**
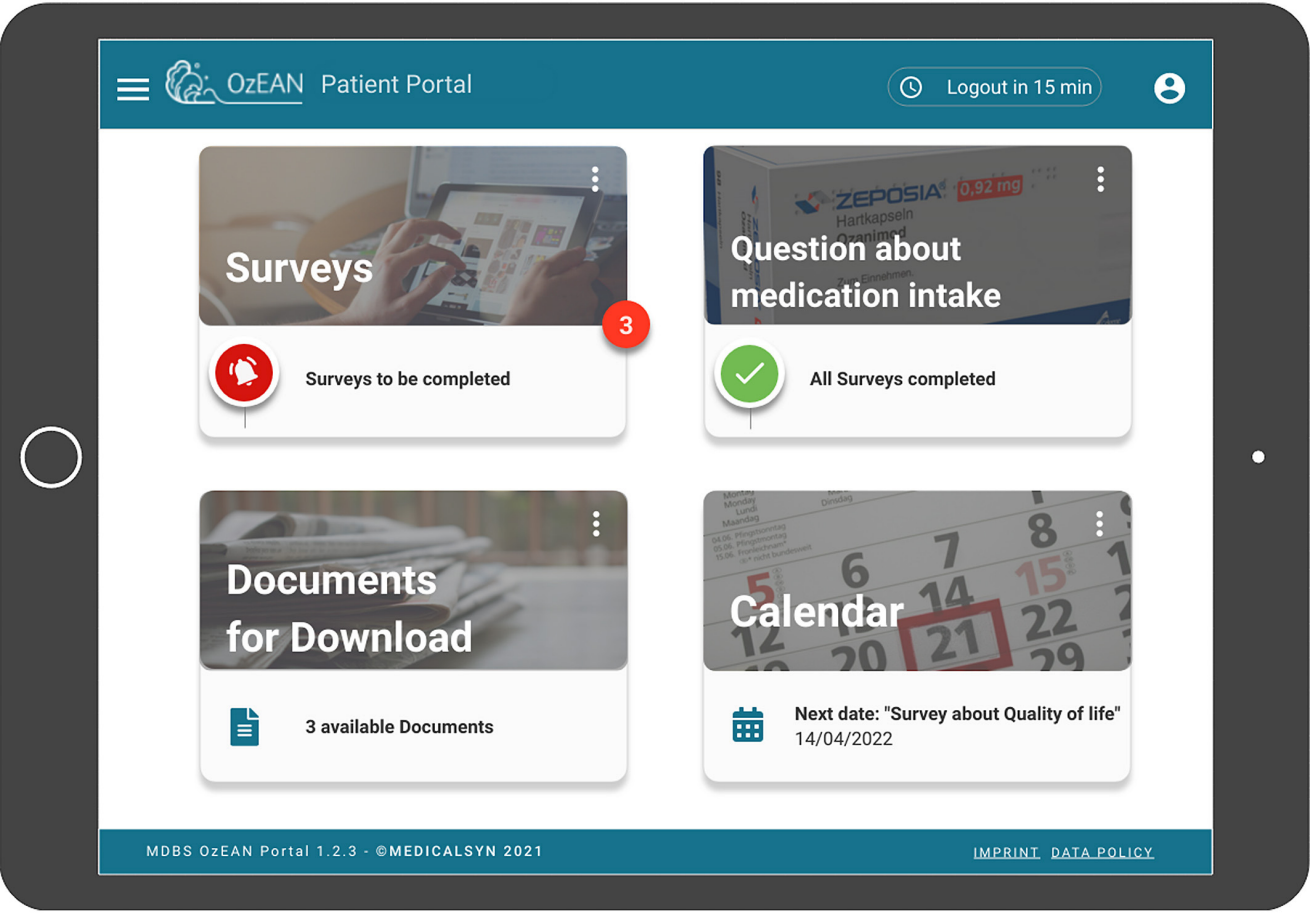
OzEAN patient portal user interface. On the start page of the patient user interface, the patient can select from the following options: surveys (PROs), questions about medication intake (medication plan), documents for download, and calendar. The medication plan displays information on ozanimod treatment. The documents module provides downloadable files (e.g., patient portal user manual and ozanimod SmPC). The calendar displays the patient's start date and participation within the OzEAN study and is equipped with a reminder to take ozanimod. The patient is also able to review and visualize personal longitudinal data graphically. PRO, patient-reported outcome; SmPC, Summary of Product Characteristics.

PROs (including adherence, TSQM v1.4, UKNDS, MSQOL-54, FSMC, WPAI-MS v2.1, and MS-HRS v3.0), preferably scored directly by the patient, are collected via the Multiple Sclerosis Documentation System 3D [MSDS^3D^ (24, 25)] with a study-specific, internet-based, patient e-health portal (Figure 3) according to the timeline in Table 2. The patient portal is accessible via computer or tablet and allows patients to download files and complete questionnaires (Figure 4). Alternatively, upon patient request, PROs are provided as paper-based versions to be completed and scored at the study site. All PRO questionnaires are made available in the German language. A detailed description of the instruments used in these assessments is available in Table 4.

**Table 4:** Description of Measures in OzEAN.

**Physician-Reported Assessment**	
Expanded Disability Status Scale (EDSS) (10)	The EDSS is a standardized, widely accepted method to evaluate disability in people with MS. Severity of disability in multiple functional systems (pyramidal, cerebellar, brain stem, sensory, bowel and bladder, visual or optic, cerebral or mental, and other) observed during a standard neurological examination is scored on a numerical scale ranging from 0 (normal) to 10 (death due to MS).
Symbol Digit Modalities Test (SDMT) (12, 13)	The SDMT is a reliable measure of change in cognitive processing speed over time. Patients are given a key showing numbers [0–9] paired with symbols. They are also presented with rows of the same symbols (in random order) and are asked to provide the matching numbers, based on the key. The score is based on the number of correct responses within 90 s, with higher scores indicating better performance. The SDMT has been validated in patients with MS and is typically administered orally in this population. Changes in SDMT raw score of ≥ 4 points or 10% are considered clinically meaningful.
**Patient-Reported Assessment**	
Treatment Satisfaction Questionnaire for Medication, version 1.4 (TSQM v1.4) (14, 15)	The TSQM v1.4 is a general measure of treatment satisfaction with medication in chronic diseases that has been tested extensively in people with RRMS. It comprises 14 items covering 4 domains: effectiveness, side effects, convenience, and global satisfaction. On individual items, patients rate their satisfaction with a medication, presence/absence and bothersomeness of side effects, extent to which side effects interfere with functioning and impact treatment satisfaction, ease of use and convenience, and confidence in the treatment. Ratings reflect experience with the medication over the previous 2–3 weeks, or since the patient's last use of the medication. Scores range from 0 to 100, with higher scores representing greater satisfaction.
United Kingdom Neurological Disability Rating Scale (UKNDS) (16, 17)	The UKNDS is a simple, user-friendly clinical disability scale that is valid and reliable for the assessment of patients with MS. It is derived from Guy's Neurology Disability Scale and consists of 11 domains: cognition, mood, vision, speech, swallowing, upper limb function, lower limb function, bladder function, bowel function, fatigue, and pain. Each subscale is scored on a 6-point Likert scale ranging from 0 (normal) to 5 (total loss of function/maximum impairment), producing an overall sum score ranging from 0 (best) to 55 (worst). Improvement or worsening of at least 1 grade in each subscale are considered clinically meaningful.
Multiple Sclerosis Quality of Life-54 (MSQOL-54) (18)	The MSQOL-54 is a multidimensional health-related QoL measure that combines both generic and MS-specific items into a single instrument. The generic component is the Short Form-36 Health Survey (SF-36) (42), to which 18 items were added to identify MS-specific issues. There are 54 items distributed into 12 subscales (physical function, role limitations-physical, role limitations-emotional, pain, emotional well-being, energy, health perceptions, social function, cognitive function, health distress, overall quality of life, and sexual function), along with 2 summary scores (physical composite summary [PCS] and mental health composite summary [MCS]). A change in score equivalent to 0.5 SD has been found to have almost universal relevance as a minimum clinically important difference for health-related QoL. Using the 0.5 SD threshold for the SF-36, a change of ≥ 5 points in PCS and MCS has been proposed to be clinically meaningful (23).
Fatigue Scale for Motor and Cognitive Functions (FSMC) (19)	The FSMC includes a cognitive scale and a physical (motor) scale, each consisting of 10 items. Items are scored using a 5-point scale ranging from 1 to 5, yielding a total score range of 20 (no fatigue at all) to 100 (severest grade of fatigue). Based on cut-off values for severity categories, a change from baseline of ≥ 10 in FSMC sum score, ≥ 6 in cognitive subscore, and ≥ 5 in physical subscore should denote a clinically meaningful change.
Work Productivity and Activity Impairment Questionnaire for Multiple Sclerosis (WPAI-MS v2.1) (20)	The WPAI-MS German v2.1 consists of 6 items across 4 domains: absenteeism (work time missed), presenteeism (impairment at work/reduced on-the-job effectiveness), work productivity loss (overall work impairment/absenteeism + presenteeism), and activity impairment. Each domain is measured on a scale of 0% to 100% impairment. The recall period is 7 days. A lower score on the WPAI-MS v2.1 subscales indicates less impairment (i.e., an improvement).
Multiple Sclerosis Health Resource Survey (MS-HRS v3.0) (21)	The MS-HRS v3.0 is a validated, 24-item questionnaire that enables a holistic and longitudinal examination of resource use and costs (direct medical, direct non-medical, and indirect) in patients with MS. It documents social resource use, independent of source of reimbursement, and economic impact on work, family, and leisure. The instrument allows for allocation of a monetary value to a specific disease state, to an event (e.g., a relapse), or to a specific therapy.

*MS, multiple sclerosis; QoL, quality of life; RRMS, relapsing-remitting multiple sclerosis; SD, standard deviation*.

AEs elicited as part of PRO data collection are subject to AE reporting. Investigators must review responses provided by patients to PRO instrument questions that assess safety to determine if AE reporting is warranted. The physician will enter AE data into the eCRF.

In the safety follow-up visit for patients withdrawn from the observational study before month 60, safety-related data (AEs) and data on any subsequent MS treatment and concomitant medication are collected in addition to PROs.

#### Data Management

Documentation of study data by physicians and authorized site staff is done exclusively online using the eCRF. The data are transmitted via secure connections and stored on secure servers of MedicalSyn GmbH, the eCRF, data management, and patient portal provider. Data entries in the patient portal are checked for plausibility and accuracy using validation programs, which generate automated queries. Open queries are displayed in the current status overview to be resolved. Data management screens new or updated free text entries in the patient portal for hidden AEs. The system follows open queries on a regular basis and communicates queries to the site. The site responds to open queries online.

#### Statistical Methods

According to the non-interventional design of the study, the statistical analyses are descriptive and exploratory. No statistical hypotheses are formulated. There will be no inferential testing, and no *P* value will be provided. Summary statistics for continuous variables will include number of observations available and number of missing values, minimum, maximum, median, mean, and standard deviation. Summary statistics for discrete variables will be presented with counts and percentages and number of missing variables.

The analysis set for the primary analysis will comprise all patients who received at least 1 dose of ozanimod during the study and for whom at least 1 postbaseline documentation is available. A subgroup analysis will be performed examining pretreatment with disease-modifying therapies (i.e., patients who are treatment-naive compared with patients who switched from another disease-modifying therapy to ozanimod).

If a patient is lost to follow-up, efforts will be undertaken to collect the data from the previous visit. Data management procedures will be implemented to limit the amount of non-reported data. Analysis methods for handling missing data (e.g., last observation carried forward, imputation, or sensitivity analyses) will be applied.

Treatment (e.g., dosage, duration of treatment, dose modifications, treatment interruptions, and concomitant medications) will be analyzed descriptively.

The analysis of the primary endpoint of persistence rate over 60 months in routine clinical practice will be calculated descriptively as percentage value at 60 months (i.e., the proportion of patients who are on continuous treatment with ozanimod at this time point), including corresponding 95% CIs calculated by the Clopper-Pearson method. A patient will be classified as non-persistent if a medication gap > 90 days occurs before the end of the 60-month documentation period. A sensitivity analysis will be performed evaluating patients who are lost to follow-up as “non-persistent.” The persistence rate will be evaluated by Kaplan-Meier methods over the entire study period (patients who are lost to follow-up will be right-censored).

The analysis of persistence rate at months 12, 24, 36, and 48 (secondary endpoint) will be calculated descriptively and summarized as the proportion of patients on continuous treatment at the respective time point, with corresponding 95% CIs.

Medication adherence (secondary endpoint) will be evaluated categorically in terms of the percentage of doses taken as prescribed.

Secondary endpoints of clinical effectiveness (ARR, EDSS, and SDMT) and all PRO measures (changes from baseline, including proportion of patients who achieved a clinically meaningful change from baseline) will be analyzed descriptively. For TSQM v1.4, it is assumed that a clinically meaningful relationship between TSQM v1.4 domains and clinical outcomes is given if the effect size (Cohen's d) is > 0.3 (15).

All AE data will be listed and summarized. AEs will be classified using the Medical Dictionary for Regulatory Activities (MedDRA) classification system. The incidences based on the patient population enrolled as well as incidence density rates (number of events/sum of person-time in years) of all AEs, serious AEs (SAEs), adverse drug reactions (ADRs), and serious ADRs will be summarized by system organ class, preferred term, and relationship to study treatment. AEs leading to discontinuation from treatment will also be summarized and listed separately.

SAS software version 9.2 or higher will be used for analyses.

### Monitoring

An external steering committee is planned for data review, data evaluation, and publication in agreement with the leading principal investigator.

An interim analysis will be conducted after enrollment of 25% of the planned number of patients and will describe the baseline data. Thereafter, yearly interim analyses are planned.

AEs are collected during study site visits and via the patient portal, as described previously. All AEs occurring after the first dose of ozanimod until the end of the study (including the safety follow-up), whether related or not to ozanimod treatment, will be recorded and reported to the sponsor or its designee. AEs and SAEs are reported within 24 h. If it is discovered that a patient or female partner of a male patient is pregnant, this is reported to the sponsor within 24 h. SAEs associated with the pregnancy are reported within 24 h. Follow-up information on pregnancy outcomes is forwarded to the sponsor, even if the outcome becomes known after the end of the study.

Representatives of the sponsor and/or its delegates are permitted to visit all study site locations to assess the data quality and study integrity. On site, they review study files and, if allowed by local laws and regulations, patient medical charts to compare them with source documents, discuss the conduct of the study with the investigator, and verify that the facilities remain acceptable. In addition, the study may be evaluated by the sponsor's internal auditors and government inspectors, who are permitted access to CRFs, source documents, other study files, and study facilities.

## Discussion

OzEAN is an ongoing study to evaluate ozanimod treatment for RRMS in real-world clinical practice in Germany, with a large sample size, sophisticated methodology, and complex design involving endpoints related to multiple MS symptoms and functional consequences. The findings from this real-world evidence study will provide an important complement to the efficacy and safety results from highly structured randomized controlled trials (26), including phase 3 clinical trials (6, 7) and the ongoing open-label extension study of phase 1–3 trials (DAYBREAK) (27).

The eligibility criteria of OzEAN are less selective than those of the phase 2 and 3 clinical trials, allowing for inclusion of adults (≥18 years) of any age with RRMS who are eligible for ozanimod treatment and elect to initiate ozanimod treatment prior to and independent from study enrollment. There are no requirements in terms of relapse history, EDSS score, MRI parameters, or prior MS treatment. This is beneficial in 2 respects. First, it will be important to establish the efficacy and safety of ozanimod in a heterogeneous and extended population outside of the highly regulated confines of a clinical trial, in which patients are selected based on predetermined criteria that may not be met by a majority of patients in clinical care (28). Second, this will allow for characterization of patients prescribed ozanimod in real-world clinical practice. A question of interest is whether ozanimod is prescribed to patients newly diagnosed with MS, consistent with a shift toward earlier use of high-efficacy disease-modifying therapies (29), or reserved for patients previously treated with lower-efficacy agents (i.e., an escalation approach) (30).

The OzEAN study incorporates assessments of persistence and adherence to treatment. Persistence (remaining on continuous treatment) and adherence (taking treatment as prescribed) are important to treatment outcomes but tend to be poor in people with MS (31). Generally, persistence and adherence are better with oral disease-modifying therapies compared with injectables, but there are differences even among oral medications that can impact treatment success (31, 32). Assessment of persistence and adherence to ozanimod treatment is an important element of the OzEAN study and complements the TSQM v1.4 as another index of treatment satisfaction.

The efficacy outcome measures of the OzEAN study complement those of the phase 2 and 3 clinical trials (Table 5), which focused on traditional, physician-reported assessments of relapses (ARR) and disability progression (EDSS), MRI parameters (gadolinium-enhancing lesions, T2 lesions, and brain atrophy), and functional measures [the Multiple Sclerosis Functional Composite (MSFC)(33)), which comprises the Timed 25-Foot Walk (lower limb function and walking speed), Nine-Hole Peg Test (upper limb function and dexterity), and either the SDMT or the Paced Auditory Serial Addition Test (cognition) (4, 6, 7, 34, 35). QoL was assessed in the phase 3 studies using the MSQOL-54, but no other PROs were employed (6, 7). The value of assessing symptoms and consequences of MS that are viewed as important by patients, as well as the patient's perspective on treatment outcome and success, is increasingly recognized (26). The OzEAN study employs a number of PROs designed to evaluate not only QoL (MSQOL-54) and cognition (SDMT), but also treatment satisfaction (TSQM v1.4), patient-assessed disability (UKNDS), fatigue (FSMC), functioning at work and in other contexts (WPAI-MS v2.1), and costs associated with MS (MS-HRS v3.0). These PROs are valid, reliable, and responsive to change, with established thresholds for clinically meaningful change (Table 4).

**Table 5:** Outcome measures in OzEAN compared with phase 2 and phase 3 studies of ozanimod.

	**Phase 2 (4)**	**Phase 3 SUNBEAM (7)**	**Phase 3 RADIANCE (6)**	**OzEAN**
**Clinical**				
ARR	X	X	X	X
Disability progression (EDSS)		X	X	X
MSFC		X	X	
T25FW		X	X	
9HPT		X	X	
SDMT		X		X
PASAT			X	
Persistence with therapy				X
**MRI**				
Gadolinium-enhancing lesions	X	X	X	X^a^
New or enlarging T2 lesions	X	X	X	X^a^
Brain atrophy		X	X	
**Patient-reported outcomes**				
MSQOL-54		X	X	X
TSQM v1.4				X
UKNDS				X
FSMC				X
WPAI-MS v2.1				X
MS-HRS v3.0				X
Adherence to therapy				X
**Safety and tolerability**				
AEs	X	X	X	X
Laboratory values	X	X	X	X^a^

*9HPT, Nine-Hole Peg Test; AE, adverse event; ARR, annualized relapse rate; EDSS, Expanded Disability Status Scale; FSMC, Fatigue Scale for Motor and Cognitive Functions; MRI, magnetic resonance imaging; MSFC, Multiple Sclerosis Functional Composite; MS-HRS v3.0, Multiple Sclerosis Health Resource Utilization Survey, version 3.0; MSQOL-54, Multiple Sclerosis Quality of Life-54; PASAT, Paced Auditory Serial Addition Test; SDMT, Symbol Digit Modalities Test; T25FW, Timed 25-Foot Walk; TSQM v1.4, Treatment Satisfaction Questionnaire for Medication, version 1.4; UKNDS, United Kingdom Neurological Disability Rating Scale; WPAI-MS v2.1, Work Productivity and Activity Impairment Questionnaire for Multiple Sclerosis, version 2.1*.

a *Only if available based on the local clinical routine assessments performed at the respective study site*.

To our knowledge, OzEAN is the first non-interventional study in MS to offer a patient portal. While use of the MSDS^3D^ data collection and management system has been incorporated into other real-world studies in MS (36–40), the expansion of this tool to include patient connection via the portal is intended to facilitate study participation, optimally inform patients, and support study patients' compliance. The aim of the patient portal is to obtain a high-resolution picture of the course of the disease with the highest possible data quality, independent from visits, through at-home documentation of digital PRO questionnaires (41). The patient portal complements and optimizes medical care in daily practice, as it enables the patient to play an active role in the study and to increase the e-health interaction between patient and study center.

This study has a number of further strengths. It represents the first collection of real-world data from patients with RRMS initiating treatment with ozanimod according to the SmPC. The results will broaden the understanding of ozanimod's safety and efficacy outside of controlled clinical conditions and in a patient population chosen based only on criteria outlined in the SmPC, and it will provide needed information to physicians and other health care providers on ozanimod treatment in routine clinical care. A high level of external validity can be expected, as the study sites and patient sample are selected to be representative of treatment and care in Germany.

There are also limitations. As OzEAN is an observational study, there is no randomization of patients, no blinding, and no control group. Outcome measures are limited to clinical assessments and PROs, with no MRI endpoints. Patient recall bias is a possible limitation, particularly with the long (6-month) intervals between assessments in the latter portion of the study. There is potential for selection bias with regard to participating sites and patients, as well as attrition bias, but measures are taken to minimize these issues, including participation of a large number of randomly selected sites of different types and locations across Germany, a large sample size, consecutive enrollment of eligible patients, and prespecified methods for handling missing data from patients lost to follow-up. Finally, the generalizability of the findings may be limited, as all of the study sites are located in Germany, a technologically advanced and predominantly racially/ethnically homogeneous country with a universal healthcare system.

## Conclusion

In summary, this is the first long-term, real-world study of ozanimod in patients with RRMS. These data will add to the safety and efficacy profile of ozanimod previously demonstrated in the phase 3 trials (6, 7) and the ongoing open-label extension study of phase 1–3 trials (DAYBREAK) (27) in patients with relapsing MS, and they will provide new information on endpoints not previously evaluated in ozanimod clinical trials. To our knowledge, this is the first non-interventional study utilizing a patient portal, which is expected to facilitate study participation and compliance, provide valuable information on PROs, and draw a high-resolution picture of the course of disease independent of study visits in a convenient way (41). Final long-term results are anticipated after study completion in March 2029; yearly interim analyses are planned after enrollment has reached 25%. The OzEAN study aims to assess the utility of ozanimod in clinical practice.

## Ethics and Dissemination

The required approvals from Ethics Committees, Independent Review Committees, Regulatory Authorities, and/or other local governance bodies were obtained before study initiation. The observational plan, patient questionnaires, and informed consent forms were reviewed and approved by the Independent Ethics Committee. The study was disclosed to the higher federal authority, to the German Association of Statutory Health Insurance Physicians, to the Central Federal Association of the Health Insurance Fund, and to the Association of Private Health Insurance Funds as required by §67 (6) German drug law. In accordance with local regulations, all patients provide written consent before enrollment. Investigators ensure that patients or their legally acceptable representatives are clearly and fully informed about the purpose of the study, potential risks, and the patient's rights and responsibilities when participating in this study.

## Author Contributions

Study design: TZ, BK, A-MP, ML, and RL. Study investigator: MB, TZ, and MM. Enrolled patients: TZ, SR, MM, and MB. Collection and assembly of data: CRO MedicalSyn. Data interpretation, manuscript preparation, manuscript review and revisions, and final approval of manuscript: All authors.

## Funding

This study received funding from Bristol Myers Squibb. The funder had/will have the following involvement with the study: contributions to study design and data analysis; decision to publish, in agreement with the Steering Committee; and provision of funding for manuscript preparation.

## Conflict of Interest

TZ: personal compensation and project support from Alexion, Almirall, Biogen, Bristol Myers Squibb, Janssen, Novartis, Roche, Sanofi, and Teva. SR: travel grants and honoraria for consulting from Biogen, Bristol Myers Squibb, Janssen, Merck, Sanofi, and Roche. MM: received compensation for activities with Almirall, Biogen, Celgene, Genzyme, Janssen, Merck, Novartis, Roche, and Sanofi, as well as research support from Merck. MB: honoraria for lecturing, consulting, and/or travel expenses for attending meetings from Bayer, Biogen, Boehringer, Bristol Myers Squibb, Coloplast, Daiichi-Sankyo, Das Fortbildungskolleg, Merck, Novartis, Roche, Sanofi, and Teva. BK: employee and works on behalf of Bristol Myers Squibb GmbH & Co. KGaA, Munich, Germany. A-MP: employee and works on behalf of Bristol Myers Squibb GmbH & Co. KGaA, Munich, Germany. ML: employee and works on behalf of Bristol Myers Squibb GmbH & Co. KGaA, Munich, Germany. RL: received compensation for activities with Biogen, Celgene, Genzyme, Janssen, Merck, Novartis, and Roche, as well as research support from Novartis.

## Publisher's Note

All claims expressed in this article are solely those of the authors and do not necessarily represent those of their affiliated organizations, or those of the publisher, the editors and the reviewers. Any product that may be evaluated in this article, or claim that may be made by its manufacturer, is not guaranteed or endorsed by the publisher.
